# Effects of human antimicrobial cryptides identified in apolipoprotein B depend on specific features of bacterial strains

**DOI:** 10.1038/s41598-019-43063-3

**Published:** 2019-04-30

**Authors:** Rosa Gaglione, Angela Cesaro, Eliana Dell’Olmo, Bartolomeo Della Ventura, Angela Casillo, Rocco Di Girolamo, Raffaele Velotta, Eugenio Notomista, Edwin J. A. Veldhuizen, Maria Michela Corsaro, Claudio De Rosa, Angela Arciello

**Affiliations:** 1 Department of Chemical Sciences, University of Naples Federico II, 80126 Naples, Italy; 2 Department of Physics, University of Naples Federico II, 80126 Naples, Italy; 3 Department of Biology, University of Naples Federico II, 80126 Naples, Italy; 4 Department of Infectious Diseases and Immunology, Division Molecular Host Defence, Faculty of Veterinary Medicine, Utrecht University, Utrecht, The Netherlands; 5 Istituto Nazionale di Biostrutture e Biosistemi (INBB), Rome, Italy

**Keywords:** Peptides, Antimicrobial resistance

## Abstract

Cationic Host Defense Peptides (HDPs) are endowed with a broad variety of activities, including direct antimicrobial properties and modulatory roles in the innate immune response. Even if it has been widely demonstrated that bacterial membrane represents the main target of peptide antimicrobial activity, the molecular mechanisms underlying membrane perturbation by HDPs have not been fully clarified yet. Recently, two cryptic HDPs have been identified in human apolipoprotein B and found to be endowed with a broad-spectrum antimicrobial activity, and with anti-biofilm, wound healing and immunomodulatory properties. Moreover, ApoB derived HDPs are able to synergistically act in combination with conventional antibiotics, while being not toxic for eukaryotic cells. Here, by using a multidisciplinary approach, including time killing curves, Zeta potential measurements, membrane permeabilization assays, electron microscopy analyses, and isothermal titration calorimetry studies, the antimicrobial effects of ApoB cryptides have been analysed on bacterial strains either susceptible or resistant to peptide toxicity. Intriguingly, it emerged that even if electrostatic interactions between negatively charged bacterial membranes and positively charged HDPs play a key role in mediating peptide toxicity, they are strongly influenced by the composition of negatively charged bacterial surfaces and by defined extracellular microenvironments.

## Introduction

Antimicrobial peptides (AMPs) are effectors of the innate immune system in a wide variety of species from the plant and animal kingdoms, including humans^1^. Although structurally different, most of these peptides fold into amphiphilic structures due to their short size (<50 amino acid residues), net positive charge and high content of hydrophobic residues^2^. Their activity against a wide range of microorganisms combined with their unique property of displaying few to no resistance effects^3^ allowed AMPs to gain great attention as promising and effective alternatives to conventional antibiotics, also against strains resistant to approved antibacterial agents^4^. Since these peptides are also able to modulate the immune response of host organisms, their efficiency is considerably enhanced. Because of this extension of functionalities, they have been more properly named “host defence peptides” (HDPs)^5,6^. The key features that make HDPs antimicrobial are their cationic nature, their ability to bind to bacterial membranes and to adopt specific secondary structures in membrane environments^7^, an essential prerequisite to their attachment and insertion into bacterial membranes. HDPs have been found to kill bacteria by first associating with their negatively charged cell surfaces and subsequently disrupt their cell membranes *via* mechanisms that involve membrane thinning, formation of transient pores, or disruption of lipid matrix, which impairs barrier function of bacterial membranes^8^. Some HDPs are also able to pass through the lipid bilayer of the membrane to act on intracellular targets^8^. Furthermore, some HDPs preferentially attack septating bacterial cells where peptides have been found to be associated to the septum and the curved regions of the outer membrane^9^. Since most HDPs target the bacterial plasma membrane directly rather than through specific protein receptors^10^, membrane phospholipid composition and net charge also play a key role in determining peptides antimicrobial activity^11^. Indeed, these parameters vary not only from bacterium to bacterium, but also as a response to changing environments^12^ and exposure to antimicrobial agents^13^. Peptide concentration also represents a further key parameter, with maximal antimicrobial activity reached only at peptide concentrations exceeding a threshold value^14^. Indeed, upon an initial electrostatic interaction between positively charged peptide molecules and negatively charged lipids, peptides reach an appropriate local concentration, allowing their penetration into the hydrophobic core of the bilayer^15,16^. Membrane bilayer thickness also appears to have an effect on the ability of a peptide to bind to the membrane and, consequently, on the ability of a lipid bilayer to induce peptide secondary structures^17,18^. However, despite decades of research, novel structural and dynamic features of membrane-associated HDPs are continuously being discovered^19^ and the exact molecular mechanism underlying HDPs ability to perturb bacterial membranes still remains controversial. Here, we analyse the antimicrobial activity of two recently characterized HDPs^20^, identified in human apolipoprotin B by using a bioinformatics method developed by our research group^21–25^. It has been reported that several eukaryotic proteins, with functions not necessarily related to host defence, act as sources of “cryptic” bioactive peptides released upon proteolytic processing by bacterial and/or host proteases^26–28^. The two novel bioactive peptides analysed in the present study represent two variants of the HDP identified in human apolipoprotein B (residues 887–922), *i.e*. peptides ApoB887–923 and ApoB887–911. These two HDPs, recombinantly produced in bacterial cells, have been here named r(P)ApoB_L_ and r(P)ApoB_S_ because of the presence of a Pro residue becoming the N-terminus of the peptides released by the acidic cleavage of an Asp-Pro bond^20^. The primary structure of the two ApoB derived HDPs is reported in Fig. S1. Both recombinant peptides have been found to be endowed with antimicrobial, anti-biofilm, wound healing and immunomodulatory properties^20^. On the other hand, they have been found to be neither toxic for mammalian cells nor hemolytic towards murine red blood cells. Interestingly, ApoB derived peptides were also found to exert significant synergistic effects in combination with either conventional antibiotics or EDTA^20^. Noteworthy, bacterial strains found to be not responsive to ApoB derived peptides, such as *S. aureus* strains and *P. aeruginosa* ATCC 27853, appeared highly susceptible to selected combinations of peptides and antibiotics or EDTA^20^, thus opening interesting perspectives to the development of successful combination therapy approaches, that have a very low potential to induce resistance phenotype. Since the definition of the molecular bases of ApoB derived peptides biological activities could greatly contribute to the rational design of effective combinatorial therapeutic approaches, in the present paper, time killing curves, Zeta potential measurements, membrane permeabilization assays, isothermal titration calorimetry studies and morphological analyses by electron microscopy have been performed.

## Results

### Killing kinetics studies

**Figure 1:**
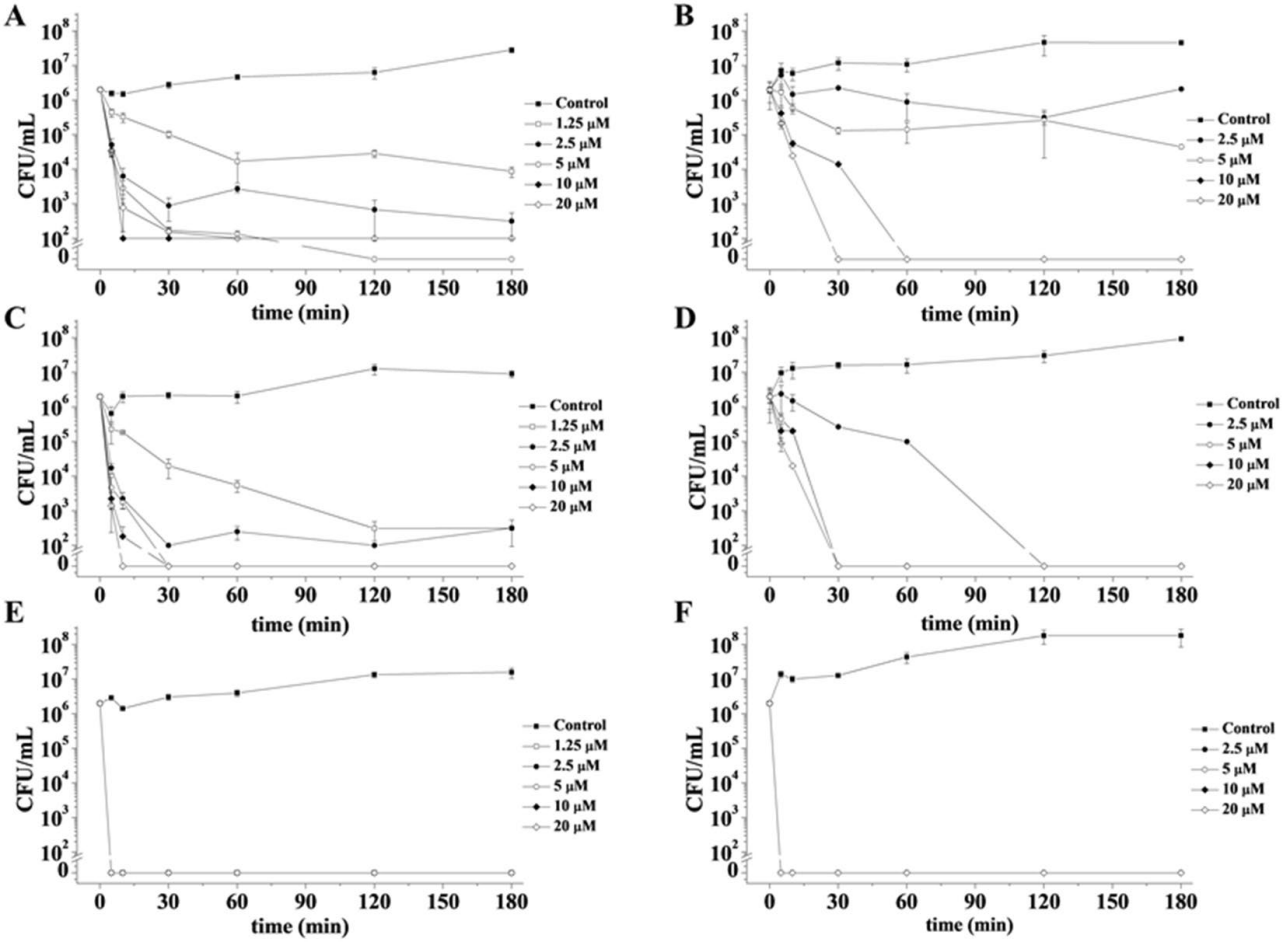
Time killing curves obtained by incubating *B. globigii* TNO BM013 (**A**,**C**,**E**) and *P. aeruginosa* PAO1 (**B**,**D**,**F**) strains with increasing concentrations of r(P)ApoB_L_ (**A**,**B**), r(P)ApoB_S_ (**C**,**D**) and CATH-2 (**E**,**F**) peptides for different lengths of time. Data represent the mean ( ± standard deviation, SD) of at least three independent experiments, each one carried out with triplicate determinations. For all the experimental points, *P < 0.05, **P < 0.01, or ***P < 0.001 were obtained for control *versus* treated samples.

It has been previously reported that ApoB derived peptides are effective on *B. globigii* TNO BM013 and *P. aeruginosa* PAO1 bacterial strains^20^, as shown in Table S1. Here, in order to analyse the kinetic of peptides bactericidal activity, we obtained kinetic killing curves by treating bacterial cells with increasing concentrations of either r(P)ApoB_L_ or r(P)ApoB_S_ for different time intervals (0–180 min). The two bacterial strains have been selected as a prototype of Gram-positive (*B. globigii* TNO BM013) and Gram-negative (*P. aeruginosa* PAO1) strains susceptible to antimicrobial ApoB derived peptides. In all the experiments, chicken cathelicidin-2 (CATH-2), a known antimicrobial peptide from chicken^29^, was tested as a positive control (Fig. 1E,F). To perform the analyses, following the incubation with peptides, control and treated samples were serially diluted and plated on agar, in order to count bacterial colonies^30^. As reported in Fig. 1A,C, at the highest peptide concentrations tested (10–20 µM), *B. globigii* TNO BM013 cells were killed within 10–30 min. At the lowest peptide concentrations (1.25–2.5 µM), instead, the same effect was obtained within 120 min (Fig. 1A,C). When peptides were tested on *P. aeruginosa* PAO1, bacterial cells were killed within 30 minutes at the highest peptide concentrations tested (10–20 µM), and within 120–180 minutes at lower peptide concentrations (2.5–5 µM) (Fig. 1B,D). In the case of CATH-2 control peptide, all the curves obtained appear perfectly superimposable, since all peptide concentrations tested were found to have the same effects. As a consequence, only the curves corresponding to the highest peptide concentration tested (20 µM) appear visible (Fig. 1E,F).

### Zeta potential measurements of bacterial cells upon treatment with peptides

**Figure 2:**
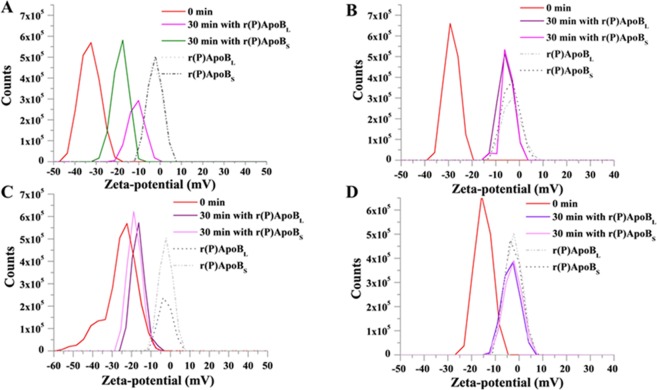
ApoB derived peptides effects on the Zeta potential of treated bacterial cells. Zeta potential values of *B. globigii* TNO BM013 (**A**) and *P. aeruginosa* PAO1 (**C**) bacterial strains were determined upon treatment with r(P)ApoB_L_ or r(P)ApoB_S_ peptides at a concentration corresponding to MIC_100_ values. Zeta potential values of bacterial strains not responsive to antimicrobial ApoB derived peptides, *i.e. S. aureus* MRSA WKZ-2 (**B**) and *P. aeruginosa* ATCC 27853 (**D**), were determined upon treatment with peptides at the highest concentration tested (40 µM) for 180 min. Dashed curves represent the Zeta potential values of ApoB derived peptides alone in bacterial culture medium (NB 0.5X). In the case of *B. globigii* TNO BM013 (**A**) and *P. aeruginosa* PAO1 (**C**) bacterial strains, a positive ζ shift is indicative of an interaction between bacterial cells and peptides. On the contrary, in the case of *S. aureus* MRSA WKZ-2 (**B**) and *P. aeruginosa* ATCC 27853 (**D**) bacterial strains, signals recorded upon treatment with peptides (continuous lines) are almost completely superimposable to those recorded for free peptide in culture medium at 40 µM (dashed lines), thus indicating absence of interactions between bacterial cells and peptides. Data represent the average of at least three independent experiments.

To evaluate ApoB derived peptides effects on bacterial membrane surface, Zeta potential (ζ) measurements were carried out. First of all, ζ values of control bacterial cells were determined over time (0–180 min) in NB 0.5X medium, in order to obtain the electrostatic potential at the shear plane of the bacteria in solution (Fig. S2). It was found that ζ did not vary throughout the incubation time in the case of all the strains tested, thus indicating their high stability. In detail, the average potential of untreated *B. globigii* TNO BM013, *S. aureus* MRSA WKZ-2, *P. aeruginosa* PAO1 and *P. aeruginosa* ATCC 27853 strains were found to be −33 ± 3, −28 ± 3, −23 ± 2, and −11 ± 2 mV, respectively (Fig. S2 and Table 1). The different values obtained for the various bacterial strains might be due to differences in membrane composition. Bacterial strains susceptible to antimicrobial ApoB derived peptides, *i.e. B. globigii* TNO BM013 and *P. aeruginosa* PAO1, were exposed to r(P)ApoB_L_ or r(P)ApoB_S_ at a concentration corresponding to their MIC values (Table S1). Upon treatment with each peptide, ζ was recorded at regular time intervals for 180 min. All the recorded ζ values are reported in Fig. S3. Values corresponding to the time point necessary to obtain complete cell death of treated bacterial cells on agar plates (30 min) have been reported for *B. globigii* TNO BM013 and *P. aeruginosa* PAO1 (both of them susceptible to antimicrobial peptides) in Fig. 2A,C, respectively. In Fig. 2B,D, instead, ζ values measured at the highest peptide concentration tested (40 µM) for *S. aureus* MRSA WKZ-2 and *P. aeruginosa* ATCC 27853 (both of them non-susceptible to antimicrobial peptides) are reported. Upon incubation of *B. globigii* TNO BM013 with r(P)ApoB_L_ or r(P)ApoB_S_, ζ was found to shift from −33 ± 3 mV to −11 ± 3 and −19 ± 3 mV, respectively (Fig. 2A and Table 1). This is indicative of the occurrence of electrostatic interactions between positively charged peptides and negatively charged bacterial surfaces, which leads to a partial neutralization of bacterial surface charge. A similar behavior was also observed in the case of Gram-negative *P. aeruginosa* PAO1 bacterial strain upon treatment with r(P)ApoB_L_ or r(P)ApoB_S_, since also in this case ζ shifted towards more positive values (Fig. 2C and Table 1). It is worth to highlight that, in the case of *B. globigii* TNO BM013 bacterial strain, a larger ζ shift was observed with respect to *P. aeruginosa* PAO1, as expected on the basis of its higher sensitivity to peptide toxicity. On the contrary, in the case of bacterial strains non-responsive to antimicrobial ApoB derived peptides, obtained signals were found to be almost completely superimposable to those recorded for the free peptide in solution at the same concentration (Fig. 2B,D, dashed lines), thus clearly indicating that only the signal attributable to the free peptide in solution was measured (Fig. 2B,D, dashed lines). This indicates that, in the case of non-responsive bacterial strains, no electrostatic interactions occur between bacterial surfaces and antimicrobial peptides. It is presumable that some extracellular factors or bacterial membrane composition might interfere with electrostatic interactions between negatively charged bacterial surfaces and positively charged peptides, and this ultimately makes peptides ineffective.

**Table 1:** Zeta-potential values recorded for bacterial cells at time 0 and upon 30 min in the absence or in the presence of ApoB derived peptides; zeta-potential values of each peptide in solution are also reported.

	SAMPLE	Z-POTENTIAL ± DS (mV)
*B. globigii* TNO BM013	Cells t_0_	−33 ± 3
	Cells t_30 min_	−29 ± 2
	Cells + r(P)ApoB_L_ t_30 min_	−11 ± 3
	Cells + r(P)ApoB_S_ t_30 min_	−19 ± 3
*P. aeruginosa* PAO1	Cells t_0_	−23 ± 2
	Cells t_30 min_	−23 ± 2
	Cells + r(P)ApoB_L_ t_30 min_	−19 ± 3
	Cells + r(P)ApoB_S_ t_30 min_	−17 ± 3
*S. aureus* MRSA WKZ−2	Cells t_0_	−28 ± 3
	Cells t_30 min_	−26 ± 2
	Cells + r(P)ApoB_L_ t_30 min_	−4 ± 3
	Cells + r(P)ApoB_S_ t_30 min_	−4 ± 3
*P. aeruginosa* ATCC 27853	Cells t_0_	−11 ± 2
	Cells t_30 min_	−10 ± 2
	Cells + r(P)ApoB_L_ t_30 min_	−3 ± 2
	Cells + r(P)ApoB_S_ t_30 min_	−3 ± 2
Peptides alone	r(P)ApoB_L_	−3 ± 1
	r(P)ApoB_S_	−3 ± 1

### Membrane permeabilization assays

To evaluate the effect of ApoB derived peptides on bacterial membrane permeability, N-Phenyl-1-naphthylamine (NPN) fluorescent probe was used. As expected, NPN uptake was found to be negligible for untreated bacterial cells, characterized by intact cell surface (Table 2 and Fig. S4A,B). The spectrofluorometric assay was also performed upon treatment of bacterial strains with ApoB derived peptides for 30 min at 37 °C. Incubation time was selected on the basis of time killing curve data (Fig. 1). *B. globigii* TNO BM013 and *P. aeruginosa* PAO1 responsive strains were treated with peptide concentrations corresponding to previously determined MIC_100_ values^20^, reported in Table S1, whereas not responsive *S. aureus* MRSA WKZ-2 and *P. aeruginosa* ATCC 27853 strains were treated with 40 µM peptides for 30 min at 37 °C. As shown in Table 2, NPN uptake factor was found to be 1.4 ± 0.9 for control *P. aeruginosa* PAO1 cells, and increased to 8.9 ± 1.2 and 11.1 ± 2.0 upon treatment with r(P)ApoB_L_ and r(P)ApoB_S_, respectively (Table 2). Similar results were obtained when responsive Gram-positive *B. globigii* TNO BM013 strain was tested. Indeed, NPN uptake factor was found to be 4.1 ± 0.9 for control cells and 7.8 ± 1.1 or 7.6 ± 1.0 for cells treated with r(P)ApoB_L_ and r(P)ApoB_S_, respectively (Table 2). On the other hand, no significant variation in NPN uptake was detected when not responsive bacterial cells were treated with ApoB derived peptides (Table 2). As a positive control, bacterial strains under test were treated with increasing concentrations of polycationic antibiotic colistin (0.25–4 μg/mL) or glycopeptide antibiotic vancomycin (0.00156–0.250 μg/mL). In both cases, following treatment, NPN uptake was found to increase in a concentration dependent manner (Fig. S4A,B). Indeed, both antibiotics, although with different mechanisms, have been reported to ultimately cause membrane permeabilization^31,32^. Altogether, obtained results confirm the crucial role played by bacterial membrane as main target of ApoB derived peptides antimicrobial activity.

**Table 2:** NPN uptake factors determined upon incubation of bacterial cells in the presence or in the absence of ApoB derived peptides.

	Samples	NPN uptake factor ± SD
*B. globigii* TNO BM013	Cells	4.1 ± 0.9
	Cells + r(P)ApoB_L_ t_30 min_	7.8 ± 1.1
	Cells + r(P)ApoB_S_ t_30 min_	7.6 ± 1.0
*P. aeruginosa* PAO1	Cells t_0_	1.4 ± 0.9
	Cells + r(P)ApoB_L_ t_30 min_	8.9 ± 1.2
	Cells + r(P)ApoB_S_ t_30 min_	11.1 ± 2.0
*S. aureus* MRSA WKZ-2	Cells t_0_	1.6 ± 0.5
	Cells + r(P)ApoB_L_ t_30 min_	0.7 ± 0.1
	Cells + r(P)ApoB_S_ t_30 min_	1.2 ± 0.1
*P. aeruginosa* ATCC 27853	Cells t_0_	0.2 ± 0.1
	Cells + r(P)ApoB_L_ t_30 min_	0.3 ± 0.1
	Cells + r(P)ApoB_S_ t_30 min_	0.5 ± 0.1

Data represent the average of at least three independent experiments.

### Morphological alterations induced by ApoB derived peptides

**Figure 3:**
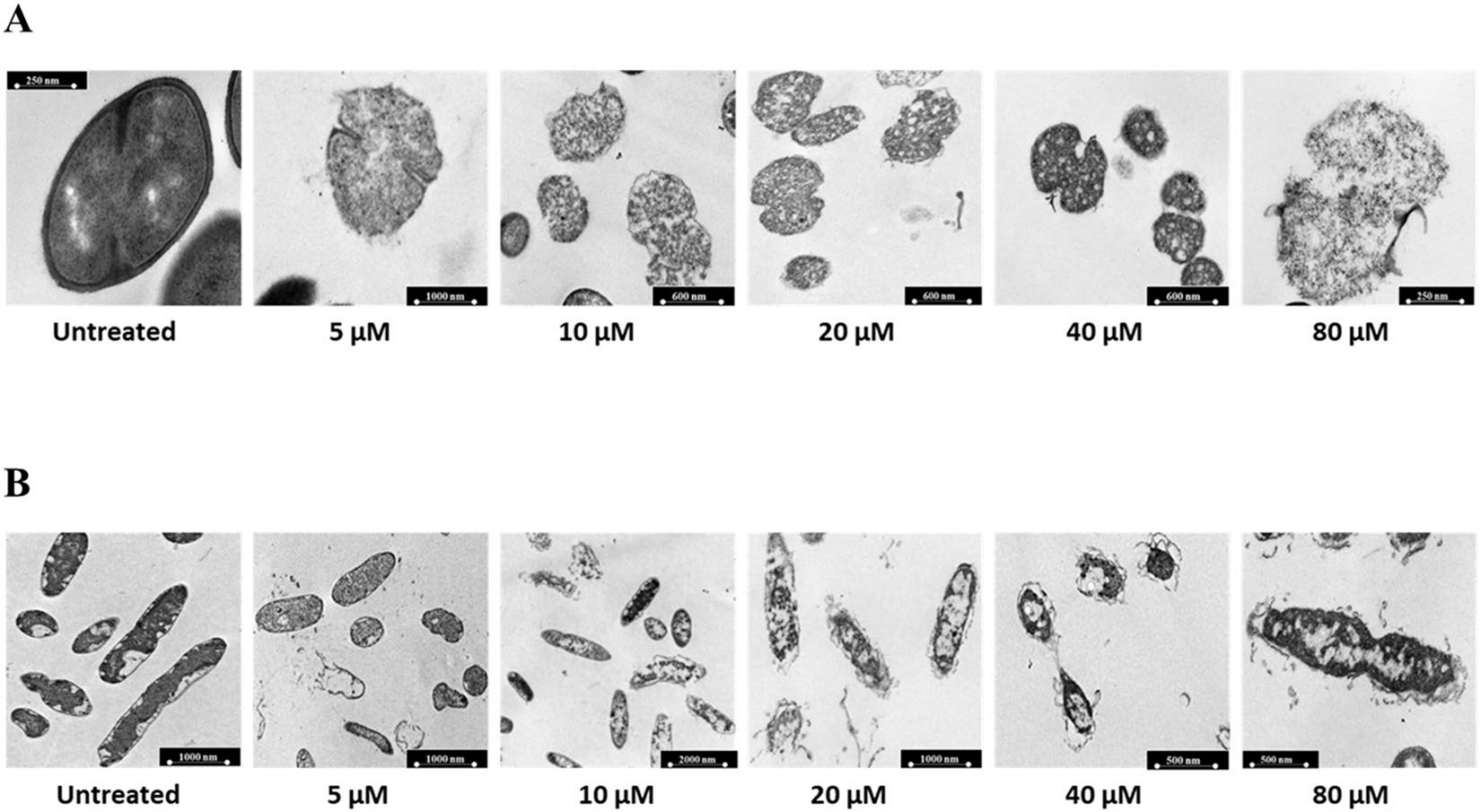
Morphological analyses of *B. globigii* TNO BM013 (**A**) and *P. aeruginosa* PAO1 (**B**) cells by TEM. Representative images are shown upon treatment of bacterial cells with increasing concentrations (0–80 μM) of r(P)ApoB_L_ peptide. A total of 60 cells were analysed for each peptide concentration in two independent experiments. Bars 250, 500 or 1,000 nm.

**Figure 4:**
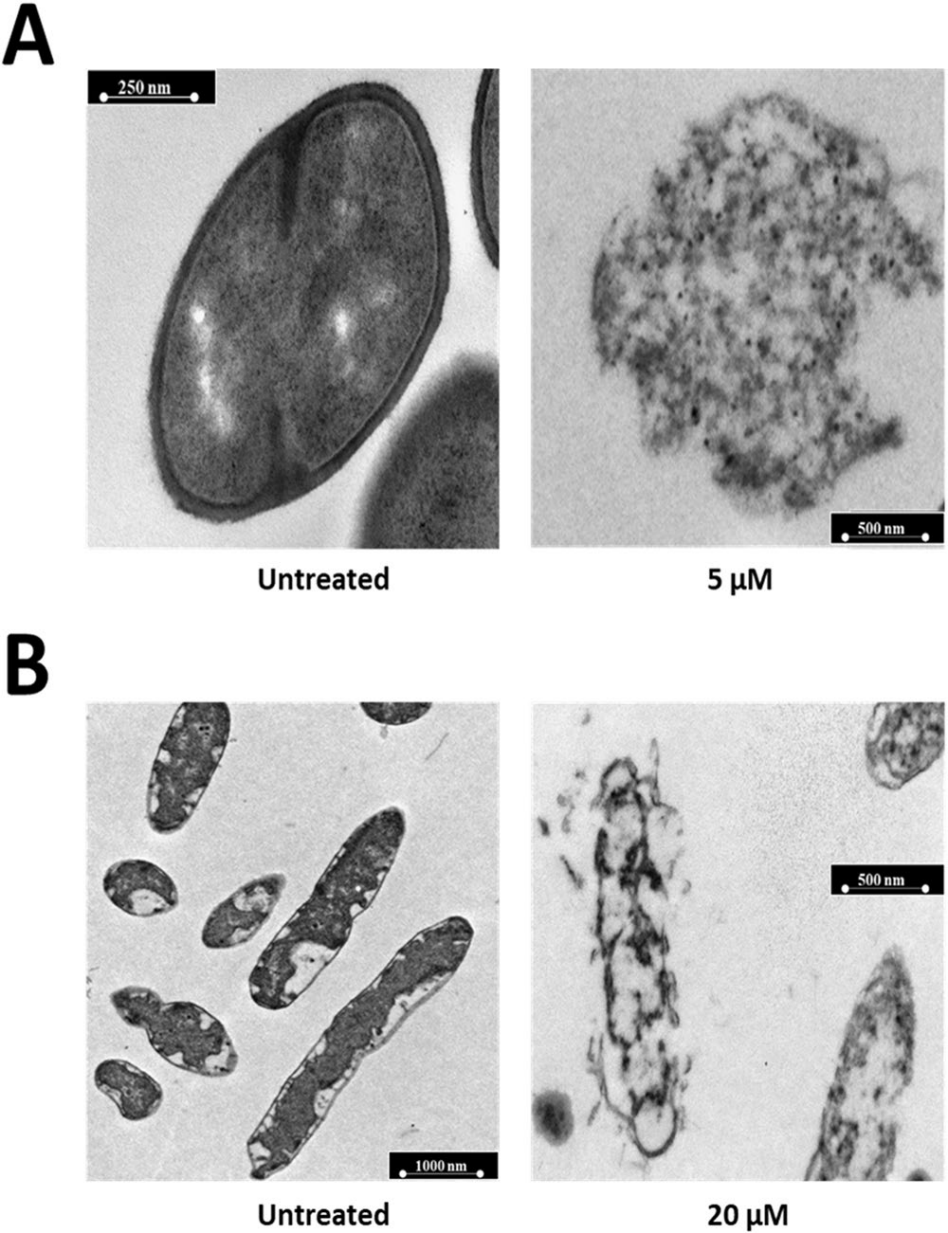
Morphological analyses of *B. globigii* TNO BM013 (**A**) and *P. aeruginosa* PAO1 (**B**) cells by TEM. Representative images are shown upon treatment of bacterial cells with r(P)ApoB_S_ concentrations corresponding to sub-MIC values (5 μM and 20 μM for *B. globigii* TNO BM013 and *P. aeruginosa* PAO1 cells, respectively). A total of 60 cells were analysed for each sample in two independent experiments. Bars 250, 500 or 1,000 nm.

To evaluate morphological alterations of bacterial strains susceptible to antimicrobial ApoB derived peptides, transmission electron microscopy (TEM) analyses were performed. In the case of control bacterial cells, intact membranes, a homogeneous intracellular distribution of DNA and ribosomes rich areas were observed (dark areas in Fig. 3). When bacterial cells were, instead, treated with increasing concentrations of r(P)ApoB_L_ peptide, a progressive detachment of cell wall and cell lysis was observed in the case of Gram-positive *B. globigii* TNO BM013 (Fig. 3A). Similarly, in the case of Gram-negative *P. aeruginosa* PAO1, a progressive wrinkling of outer membrane, dissociation of membrane fragments, permeabilization of outer and inner membranes and the leakage of electron dense material was detected (Fig. 3B). Moreover, in both cases, a complete alteration of intracellular morphology, with a decrease of cytoplasm density, was evaluated at the highest peptide concentrations tested (Fig. 3). Similar results were obtained when *B. globigii* TNO BM013 and *P. aeruginosa* PAO1 strains were treated with r(P)ApoB_S_ peptide at a concentration of 5 and 20 µM, respectively (Fig. 4A,B). It is noteworthy that significant morphological alterations were already detected when peptides were tested at sub-MIC concentrations.

**Figure 5:**
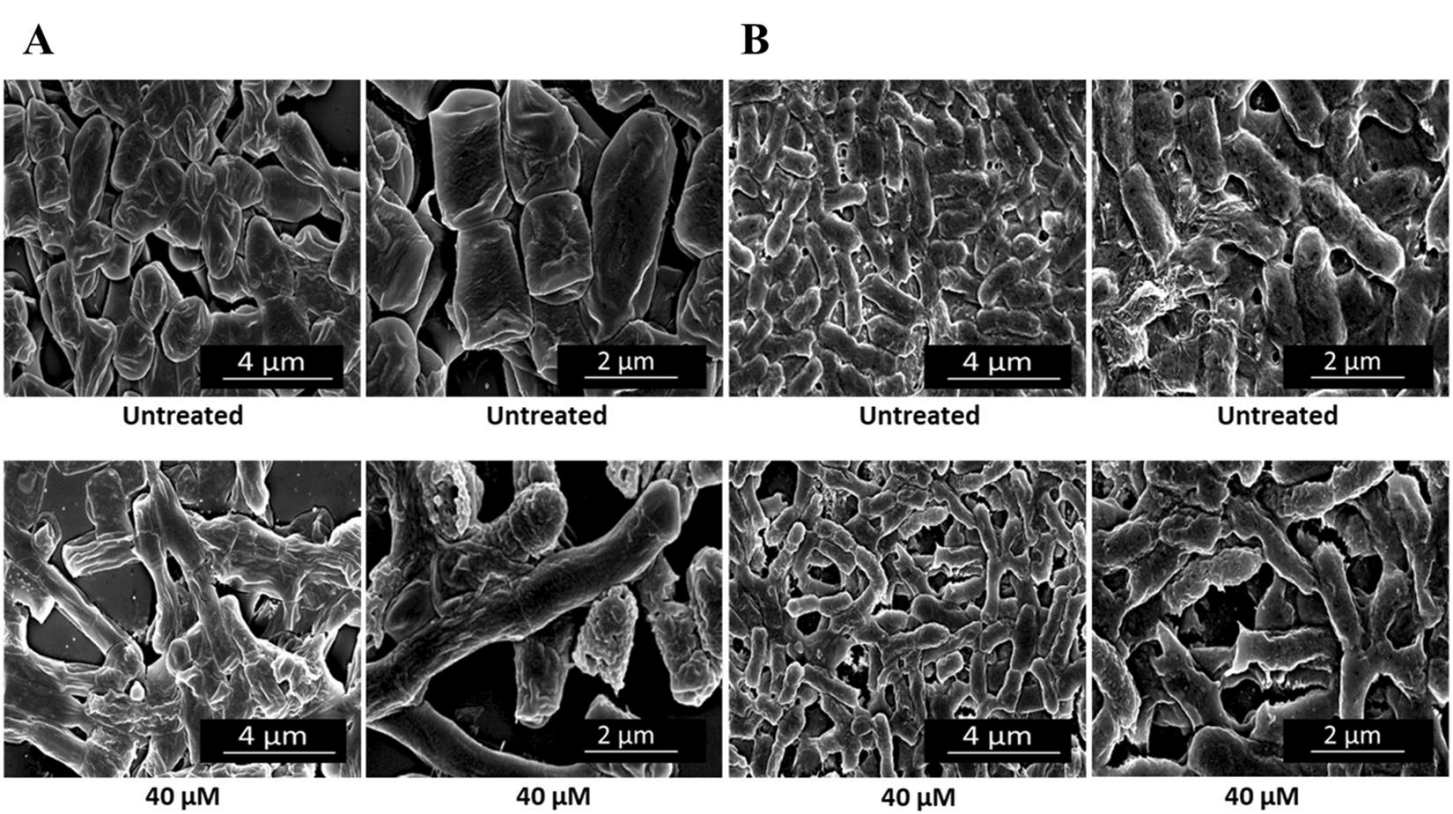
Morphological analyses of *B. globigii* TNO BM013 (**A**) and *P. aeruginosa* PAO1 (**B**) cells by SEM. Representative images are shown upon treatment of bacterial cells with 40 µM r(P)ApoB_L_. A total of 60 cells were analysed for each sample in two independent experiments. Bars 2 or 4 µm.

**Figure 6:**
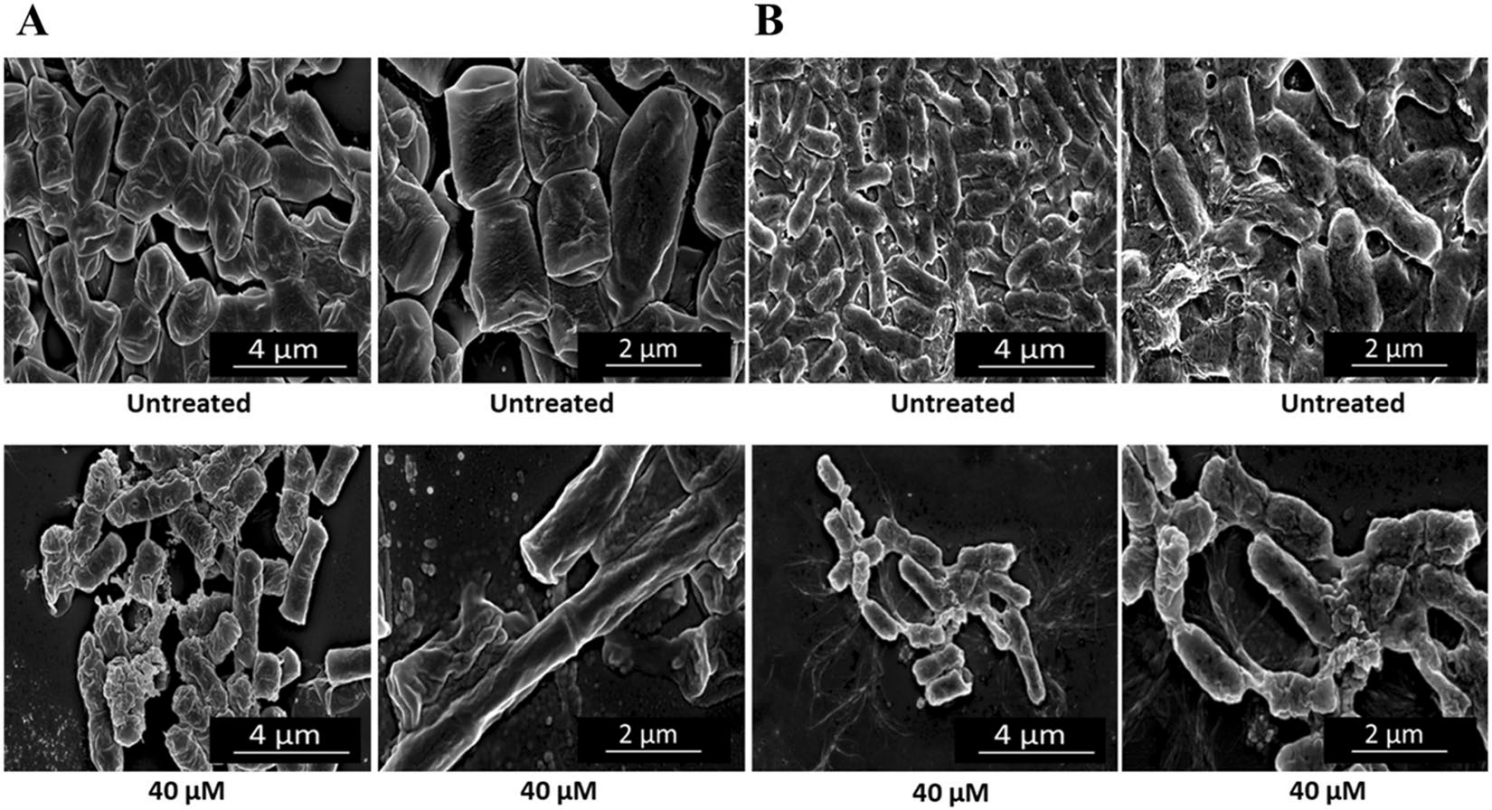
Morphological analyses of *B. globigii* TNO BM013 (**A**) and *P. aeruginosa* PAO1 (**B**) cells by SEM. Representative images are shown upon treatment of bacterial cells with 40 µM r(P)ApoB_S_. A total of 60 cells were analysed for each sample in two independent experiments. Bars 2 or 4 µm.

Furthermore, to analyse the surface of responsive bacterial cells upon treatment with antimicrobial ApoB derived peptides, scanning electron microscopy (SEM) analyses were also performed. In the absence of peptides, bacterial cells displayed smooth and intact surfaces (Figs 5 and 6). Preliminary occurrence of biofilm extracellular matrix formation was also detected in the case of control *P. aeruginosa* PAO1 cells (Figs 5B and 6B). When bacterial cells were, instead, treated with peptides, bacterial surfaces appeared corrugated with some dimples, both in the case of *B. globigii* TNO BM013 and *P. aeruginosa* PAO1 strains (Figs 5 and 6). Even more interesting is the evidence that the treatment of *B. globigii* TNO BM013 cells with ApoB derived peptides induces cells to grow as long filaments (Figs 5A and 6A), probably as a consequence of septation block, thus strongly suggesting that these peptides act by ultimately inhibiting cell division. To monitor this phenomenon, experiments were also performed by incubating *B. globigii* TNO BM013 cells with increasing concentrations of either r(P)ApoB_L_ or r(P)ApoB_S_ peptide. Obtained results, reported in Figs S5 and S6, clearly indicate that both peptides induce a septation block even at concentrations significantly lower than MIC_100_ value. Moreover, obtained results support the evidence that peptides effects strongly depend on the features of bacterial strains under test. Indeed, when *P. aeruginosa* PAO1 cells were treated with 40 µM r(P)ApoB_L_ (Fig. 5B) or 40 µM r(P)ApoB_S_ peptide (Fig. 6B), no evidence of cell division block was detected. However, in both cases, a significantly lower cell density, indicative of a massive cell death, was revealed (Figs 5B and 6B). Moreover, in the case of all the treated samples, cell surfaces appeared irregular, corrugated, and withered (Figs 5 and 6), with some dimples and signs of cytoplasm leakage especially in the case of *P. aeruginosa* PAO1strain (Figs 5B and 6B).

### Analysis of lipopolysaccharides (LPSs) isolated from *P. aeruginosa* ATCC 27853 and *P. aeruginosa* PAO1 bacterial strains

**Figure 7:**
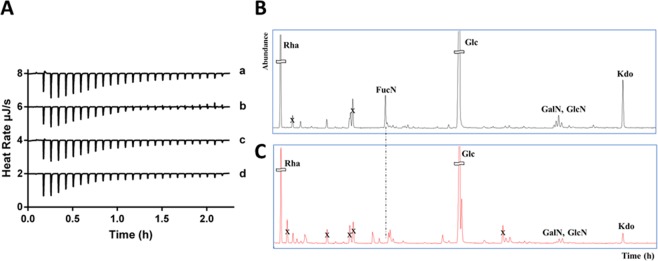
Analysis of the binding between ApoB derived peptides and LPSs extracted from *P. aeruginosa* PAO1 and *P. aeruginosa* ATCC 27853 bacterial strains by isothermal titration calorimetry (ITC) (**A**). The negative peaks indicate heat requirement for binding (endothermic), and are indicative of an entropy-driven binding reaction. (a) Binding of r(P)ApoB_L_ peptide to LPS from *P. aeruginosa* PAO1; (b) binding of r(P)ApoB_L_ peptide to LPS from *P. aeruginosa* ATCC 27853; (c) binding of r(P)ApoB_S_ peptide to LPS from *P. aeruginosa* PAO1; (d) binding of r(P)ApoB_S_ peptide to LPS from *P. aeruginosa* ATCC 27853. Chromatograms of acetylated methyl glycosides of LPSs extracted from *P. aeruginosa* PAO1 (**B**), and from *P. aeruginosa* ATCC 27853 (**C**).

We previously demonstrated, by Far UV-CD analyses, that r(P)ApoB_L_ peptide gradually assumes a defined structure in the presence of increasing concentrations of LPS, what suggests a direct binding of this peptide to LPS^20^. Here, to investigate the role played by LPS molecules in the efficacy or the inefficacy of ApoB derived peptides on Gram-negative bacterial strains, we extracted LPS molecules from *P. aeruginosa* ATCC 27853 and *P. aeruginosa* PAO1 bacteria. Afterwards, we analysed the interaction between ApoB derived peptides and the two purified LPS fractions by isothermal titration calorimetry (ITC) experiments. As shown in Fig. 7A, r(P)ApoB_L_ and r(P)ApoB_S_ peptides appear to bind to LPS molecules extracted from the two bacterial strains in a very similar fashion. Binding reactions were found to be all endothermic, thus indicating that they are driven by entropy rather than enthalpy. In the case of the binding of r(P)ApoB_L_ peptide to the LPS extracted from *P. aeruginosa* PAO1, a dissociation constant (Kd) of 3.6 × 10^−6^ M was determined, with an entropy (ΔS) of 269 J/mol and a positive enthalpy (ΔH) of 51.1 KJ/mol (Table S2). Similar spectra and binding parameters were also obtained in the case of the interaction of r(P)ApoB_S_ peptide with LPS molecules extracted from *P. aeruginosa* PA01 bacterial strain (Fig. 7A and Table S2). No significant differences were detected when the binding of both ApoB derived peptides to LPS molecules extracted from *P. aeruginosa* ATCC 27853 strain was analysed (Fig. 7A and Table S2). Altogether, obtained data indicate that both ApoB derived peptides are able to directly interact with LPS molecules extracted from the two bacterial strains, with no major differences in the binding of peptides to LPS molecules extracted from *P. aeruginosa* susceptible and not responsive bacterial strains (Fig. 7A and Table S2), at least in *in vitro* experiments and in the experimental conditions tested. To deepen on the role that LPS molecules might play in determining the different susceptibility of Gram-negative bacterial strains to ApoB derived peptides toxicity, we also performed a preliminary characterization of LPS molecules extracted from *P. aeruginosa* ATCC 27853 and *P. aeruginosa* PAO1 bacterial strains. To this purpose, LPSs extracted from dried bacterial cells by the PCP method were analysed by 14% DOC-PAGE electrophoresis and visualized by silver nitrate staining, that revealed a smooth LPS for both strains. Smooth LPS extracted from *E. coli* O55:B5 was used as a standard (data not shown). To define the glycosyl compositions of the intact LPSs, acetylated methyl glycosides were analysed by GC-MS. The monosaccharides were identified from their EI mass spectra and from their GC column retention time by comparison with authentic standards. For both LPSs, rhamnose (Rha), glucose (Glc), 2-amino-2-deoxy-D-galactopyranose (GalN), 2-amino-2-deoxy-D-glucopyranose (GlcN), and 3-deoxy-D-manno-oct-2-ulosonic acid (Kdo) were found to be present (Fig. 7B,C). The occurrence of heptoses in both LPSs was displayed only upon a de-phosphorylation reaction obtained by HF treatment (data not shown). It is important to highlight the selective presence of 2-amino-2,6-dideoxy-D-galactopyranose (FucN) in *P. aeruginosa* PAO1 LPS, as indicated by the chromatogram (Fig. 7B). It is noteworthy that the majority of *P. aeruginosa* strains have been reported to co-express two chemically and distinct forms of LPS O-antigens: (i) a serotype containing the O-antigen B-band and (ii) a common antigen referred to as O-antigen A-band. The chemical structure of the A-band from most of the sero-types was shown to be a linear α-D-rhamnan^33^; instead, the chemical structure of the B-band was reported to contain two derivatives of the 2,3-diamino-2,3-dideoxy-D-mannuronic acid (Man2N3NA) and FucN^34^. Our results suggest the absence of the B-band in the case of *P. aeruginosa* ATCC 27853 strain. This feature might be responsible for the different response of the two Gram-negative strains under test to ApoB derived peptides.

## Discussion

The healthcare burden associated to the increasing emergence of microorganisms resistant to multiple antimicrobial compounds has accelerated in recent years, and alternative weapons are urgently needed. However, despite huge efforts, the discovery, development, manufacture and marketing of new antibiotics has significantly slowed down in the past 30 years, whereas the clinical and economic impact of resistance is alarmingly rising. Although the scientific difficulty in identifying novel antibiotics may be increasing, as microbes become resistant to an ever-increasing array of treatments and, concomitantly, the identification of novel targets is challenging, the main explanation is that investments made by large pharmaceutical companies did not lead to profitable products^35^. In this scenario, naturally occurring Host Defense Peptides (HDPs) are gaining great attention. Indeed, being essential constituents of innate immunity, they represent promising lead structures to develop antibiotics against Gram-negative and Gram-positive bacteria, including strains resistant to approved antibacterial agents^4^. We recently characterized novel human HDPs identified by a bioinformatics approach in human ApoB^20,21^, and demonstrated that the two recombinant ApoB derived HDPs are endowed with a broad-spectrum anti-microbial activity, and with anti-biofilm, wound healing and immunomodulatory properties^20^. On the other hand, they have been found to be neither toxic nor hemolytic towards eukaryotic cells, what opens interesting perspectives to their therapeutic applicability^20^. However, it should be emphasized that ApoB derived AMPs were found to be antimicrobial towards four out of eight strains tested, *i.e. E. coli* ATCC 25922, *P. aeruginosa* PAO1, *B. globigii* TNO BM013, and *B. licheniformis* ATCC 21424, with MIC_100_ values comprised between 1.25 and 20 µM, indicating that they are effective on both Gram-negative and Gram-positive bacterial strains^20^. On the other hand, they were found to be ineffective towards *P. aeruginosa* ATCC 27853, methicillin-resistant *S. aureus* (MRSA WKZ-2), and *S. aureus* ATCC 29213^20^. However, when peptides were tested in combination with conventional antibiotics or EDTA, remarkable synergistic effects were detected^20^. Indeed, bacterial strains found to be not responsive to ApoB derived AMPs toxicity, such as *S. aureus* strains and *P. aeruginosa* ATCC 27853, were found to be highly susceptible to combinations of ApoB derived peptides with antibiotics or EDTA^20^. This paves the way to the development of successful combination therapy approaches, that have a very low potential to induce resistance phenotype. Since the definition of the molecular bases of ApoB derived peptides antimicrobial activity could be crucial for the design of effective combinatorial therapeutic approaches, the mechanism of action of the two ApoB derived AMPs has been here investigated by selecting four bacterial strains as prototypes of Gram-positive and Gram-negative strains susceptible or resistant to antimicrobial ApoB derived peptides. First of all, we analysed the kinetic of peptides bactericidal activity, and demonstrated that, at the highest peptide concentrations tested (10–20 µM), susceptible bacterial cells (*B. globigii* TNO BM013 and *P. aeruginosa* PAO1cells) were killed within a very short time interval, *i.e*. 30 min. This evidence is in perfect agreement with data collected by measuring Zeta potential values upon incubation of susceptible bacterial cells with ApoB derived peptides tested at concentrations corresponding to previously determined MIC_100_ values. Indeed, in all the cases, a significant increase of Zeta potential values was detected, what is indicative of the occurrence of electrostatic interactions between positively charged peptides and negatively charged bacterial surfaces, with a consequent neutralization of bacterial surface. Interestingly, a greater variation in Zeta potential values was detected in the case of the strain characterized by the highest sensitivity to peptide toxicity, *i.e. B. globigii* TNO BM013. This might be indicative of a higher electrostatic affinity between cationic peptides and negatively charged bacterial surface. Indeed, it has been reported that bacterial membrane surface neutralization is a key event mediating the anti-microbial activity of several peptides^36^, with Zeta potential alteration generally preceding membrane permeabilization leading to cell death^36^. In perfect agreement with this finding, a significant increase of NPN uptake factor was selectively detected upon treatment of susceptible bacterial strains with ApoB derived peptides, thus strongly supporting the evidence that Zeta potential increase precedes membrane permeabilization, and confirming the crucial role played by bacterial membrane as main target of ApoB derived peptides antimicrobial activity. Moreover, in the case of *B. globigii* TNO BM013 susceptible bacterial strain, Zeta potential increase and membrane permeabilization is also accompanied by a septation block, as indicated by electron microscopy analyses performed at different time intervals and upon treatment with increasing ApoB derived peptides concentrations. These findings are in perfect agreement with a recent report indicating that *E. coli* treatment with antimicrobial peptides causes cells to filament through a division block controlled by the PhoQ/PhoP signaling pathway^37^. Bacterial cell filamentation, here observed by electron microscopy analyses, might be a result of DNA replication inhibition, SOS induction, chromosome segregation, or failure of septation process^38^. It has been previously reported that treatment of *E. coli* with the antimicrobial peptide microcin J25 causes cells to filament, with a consequent inhibition of cell division processes through a non-SOS-dependent mechanism mediating a bacteriostatic mode of action^39^. Similarly, *E. coli* cells treated with diptericin showed a significantly elongated morphology, indicating that this peptide may affect cell targets involved in cell division to induce cell death, as suggested by its selective activity on actively growing *E. coli* cells^40^. Also human defensin 5 (HD5) was found to induce extensive cell elongation, with a consequent disruption of cell division events^41^. Interestingly, in agreement with our findings, such treatment outcomes were observed only upon treatment of Gram-negative bacteria with HD5, thus suggesting a common inhibitory activity depending on specific features of bacterial strains under test. Also in the case of ApoB derived peptides, effects appear to strongly depend on specific properties of analysed bacterial strains. Indeed, in the case of Gram-negative *P. aeruginosa* PAO1 strain, no signs of cell division block were detected upon treatment with ApoB derived peptides, although significant alterations of cell morphology, with irregular and corrugated cell surfaces, signs of cytoplasm leakage and cell death, were detected. It has also to be emphasized that two out of four bacterial strains selected in the present study appear resistant to ApoB derived peptides antimicrobial activity, although they are characterized by negatively charged surfaces, as indicated by Zeta potential values determinations. In particular, in the case of Gram-positive *B. globigii* TNO BM013 and *S. aureus* MRSA WKZ-2 bacterial strains, Zeta potential values have been found to be −33 ± 3 and −28 ± 3, respectively, but only *B. globigii* TNO BM013 cells were found to be susceptible to ApoB derived peptides antimicrobial activity. In the case of Gram-negative *P. aeruginosa* PAO1 and *P. aeruginosa* ATCC 27853 strains, instead, Zeta potential values were found to be very different, *i.e*. −23 ± 2 and −11 ± 2 mV, respectively. It is plausible that this dissimilarity reflects differences in bacterial membrane composition ultimately affecting peptide ability to interfere with bacterial cell viability. Since no significant effects on Zeta potential values were detected in the case of resistant bacterial cells treated with ApoB derived peptides, it has been hypothesized a failure of electrostatic interactions between negatively charged bacterial surfaces and positively charged peptides, with a consequent counteraction of ApoB derived peptides antimicrobial activity. Indeed, it has been extensively reported that electrostatic interactions play a pivotal role in the cell killing process mediated by antimicrobial peptides^42^. Based on obtained results, it has been hypothesized that some extracellular factors or bacterial membrane composition might interfere with electrostatic interactions between peptides and bacterial surfaces in the case of resistant bacterial cells. Since previous analyses by Far UV-CD suggested a direct binding of r(P)ApoB_L_ peptide to bacterial LPS^20^, experiments were here performed to verify whether ApoB derived peptides are able to directly interact with LPS molecules extracted from *P. aeruginosa* PAO1 and *P. aeruginosa* ATCC 27853 bacterial strains. Analyses, performed by isothermal titration calorimetry (ITC), indicated that both ApoB derived peptides are able to directly interact with LPS molecules extracted from the two bacterial strains, with no significant differences, at least when peptide binding to LPS molecules is tested in *in vitro* experiments. However, it has to be considered that, in physiological conditions, where up to 100,000 molecules of LPS are located at the surface of one single Gram-negative bacterium^43^, several factors, such as membrane composition or extracellular microenvironment, might interfere with peptide binding to exposed LPS molecules. Based on this, we also performed a preliminary characterization of LPS molecules extracted from *P. aeruginosa* PAO1 and *P. aeruginosa* ATCC 27853 bacterial strains, in order to evaluate whether differences in LPS structures might be responsible for the efficacy or the inefficacy of ApoB derived peptides on Gram-negative bacterial strains. Performed analyses indicated the selective presence of 2-amino-2,6-dideoxy-D-galactopyranose (FucN) in the LPS of susceptible *P. aeruginosa* PAO1 bacterial strain, whereas LPS B-band was found to be absent in the case of resistant *P. aeruginosa* ATCC 27853 strain. Since the B-band is reported to display negative charges, such as those of the Man2N3NA residues^34^, electrostatic interactions between positively charged ApoB derived peptides and LPS molecules exposed on *P. aeruginosa* PAO1 bacteria might involve these monosaccharides. Instead, the sole presence of the hydrophobic rhamnan chain (A-band) in the LPS of resistant *P. aeruginosa* ATCC 27853 strain could be responsible for the failure of the electrostatic interactions between peptides and bacterial surfaces. This might be one of the key factors responsible for the different response of the two Gram-negative bacterial strains to ApoB derived peptides toxicity.

**Figure 8:**
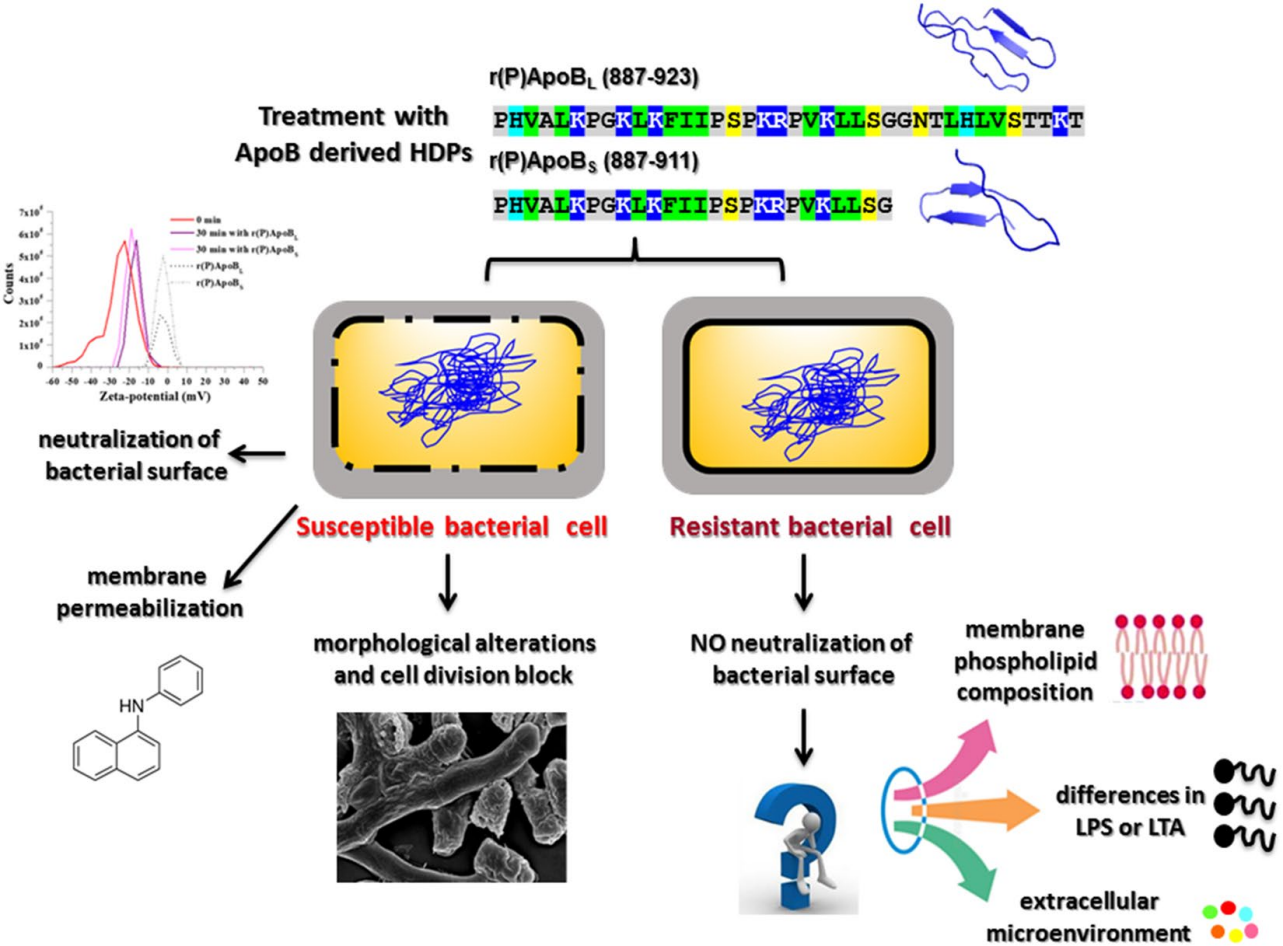
Schematic representation of the main experimental evidence herein collected.

Altogether, collected results indicate that ApoB derived peptides exert their antimicrobial activity by mainly targeting bacterial membrane and subsequently affecting intracellular molecules and/or processes, such as cell division (Fig. 8). Obtained findings also strongly indicate that an interference with electrostatic interactions between negatively charged bacterial surfaces and positively charged peptides, probably due to extracellular microenvironment or bacterial membrane composition, might counteract ApoB derived peptides antimicrobial activity (Fig. 8).

## Materials and Methods

### Materials

All the reagents were purchase from Sigma-Aldrich (Milan, Italy), unless differently specified. Chicken cathelicidin-2 (CATH-2) peptide was obtained from CPC Scientific Inc. (Sunnyvale, USA).

### Recombinant production of ApoB derived peptides

Expression and isolation of recombinant peptides was carried out as previously described^20^ with the only exception of a final gel-filtration step, that was added in order to remove salts used along the purification process and that tend to attach to the peptides, as previously reported^44,45^.

### Bacterial strains and growth conditions

Methicillin-resistant *Staphylococcus aureus* (MRSA WKZ-2), *Bacillus globigii* TNO BM013, *Pseudomonas aeruginosa* ATCC 27853, and *Pseudomonas aeruginosa* PAO1 bacterial strains were grown as previously described^20^.

### Anti-microbial activity assay

To test the anti-microbial activity of ApoB derived peptides, previously described experimental procedure was used^20^. MIC_100_ values correspond to the lowest concentration of peptide associated to no detectable bacterial growth.

### Killing kinetic studies

To kinetically analyse bacterial killing by ApoB derived peptides, experiments were performed on *B. globigii* TNO BM013 and *P. aeruginosa* PAO1 strains as previously described^20^.

### Zeta potential measurements of bacterial cells in the presence of ApoB derived peptides

To perform analyses, bacteria were grown over-night in MHB medium, then diluted in fresh MHB, and incubated at 37 °C until logarithmic phase of growth was reached. Bacteria were then diluted to 4 × 10^6^ CFU/mL in a final volume of 5 mL of NB 0.5X, and mixed with the peptide under test (1:1 v/v). In the case of bacterial strains susceptible to antimicrobial ApoB derived peptides, r(P)ApoB_L_ and r(P)ApoB_S_ were tested at concentrations corresponding to MIC_100_ values. In the case of not responsive strains, instead, only the highest peptide concentration was tested (40 µM). At defined time intervals (0–180 min), Zeta potential values were determined. Zeta potential measurements of control samples were carried out in NB 0.5X pH 7.4. To test different peptide concentrations, serial dilutions were performed, in order to mix bacteria and peptides at a ratio of 1:1 v/v. The Zeta potential of bacterial cells was determined at 25 °C from the mean of 3 independent measurements (30 runs each), in the absence and in the presence of different peptide concentrations. Zeta potential values were obtained by phase analysis light scattering (PALS) in a Zetasizer Nano ZS 90 device (Malvern, Worcestershire, UK), equipped with Helium–Neon laser (633 nm) as a source of light, with the detection at 173 degree scattering angle at room temperature (25 °C), using disposable Zeta cells with gold electrodes. Values of viscosity and refractive index were set to 0.8872 cP and 1.330, respectively.

### NPN uptake assay

NPN uptake assays were carried out by following the previously described experimental procedure^46^. To do this, 1-N-phenylnaphthylamine (NPN) was diluted to 1 mM in 5 mM HEPES buffer pH 7.2. Control wells were prepared as follows: (i) buffer alone (1 mL); (ii) buffer (1 mL) and NPN (2 µL); (iii) bacteria in buffer (1 mL); (iv) bacteria in buffer (1 mL), NPN (2 µL). Antibiotics or peptides were mixed with bacterial suspension in Eppendorf tubes for 2 and 30 min, respectively, and then transferred into cuvettes. NPN was added immediately before the measurement of fluorescence; the values were recorded within 3 min. Fluorescence emission was detected at 420 nm upon excitation at 340 nm by using a PerkinElmer LS-55 luminescence spectrometer (Waltham, MA, USA). Each assay was performed at least three times. The results are expressed as NPN uptake factors, calculated by subtracting background, *i.e*. the value obtained in the absence of NPN.

### Transmission electron microscopy

Bactericidal effects of ApoB derived peptides were also investigated by transmission electron microscopy (TEM) analyses, which required higher bacterial cell densities (2 × 10^8^ CFU/mL). For this reason, additional colony count assays were performed to determine ApoB derived peptides MIC_100_ values at this bacterial cell density. In the case of both peptides, a MIC_100_ value of about 80 μM was determined. To perform analyses, *B. globigii* TNO BM013 and *P. aeruginosa* PAO1 strains were incubated with increasing concentrations of peptides (0–80 μM) for 3 hrs at 37 °C. Samples were then fixed with 2% glutaraldehyde (Polysciences, Eppelheim, Germany) in 5 mM CaCl_2_, 10 mM MgCl_2_ (both Merck, Darmstadt, Germany) in 0.1 M sodium cacodylate buffer pH 7.4 over-night at 4 °C. Bacteria treatment with peptides was stopped by adding the fixative and keeping the cells over-night at 4 °C. Cells were then washed 3 times by incubation in sodium cacodylate buffer for 10 min and embedded in 2% low-melting point agarose v/v. Cells were then post-fixed with 4% osmium tetroxide (Electron Microscopy Sciences, EMS, Hatfield, USA) and 1.5% K_4_Fe(CN)_6_–3H_2_O (Merck, Darmstadt, Germany) in distilled water for 2 hrs at 4 °C. Upon cell washing with distilled water (5 times for 10 min), cells were incubated in 0.5% uranylacetate (EMS, Hatfield, USA) for 1 hr at 4 °C. After further washing with distilled water (3 times for 10 min), samples were embedded in Epon resin and ultrathin sections (50 nm) of each block were prepared by using a Leica UCT ultramicrotome (Leica, Vienna, Austria). Obtained sections were stained with uranyl acetate and lead citrate by using the Leica AC20 system (Leica, Vienna, Austria). TEM images have been acquired in bright field mode using a Philips EM 208 S transmission electron microscope with an accelerating voltage of 80 kV.

### Scanning electron microscopy

Bacterial cells in exponential growth phase were grown for 3 hrs in NB 0.5X in microfuge tubes in the absence or in the presence of ApoB derived peptides. Also in this case, high bacterial cell densities were required (2 × 10^8^ CFU/mL) for the analysis, and it should be underlined that, in these experimental conditions, peptides MIC_100_ value was found to be about 80 μM. To perform scanning electron microscopy (SEM) analyses, *B. globigii* TNO BM013 and *P. aeruginosa* PAO1 strains were incubated with 40 μM peptides for 3 hrs at 37 °C. Following incubation, bacterial cells were centrifuged at 10,000 rpm at 4 °C and fixed in 2.5% glutaraldehyde. Following over-night incubation, bacterial cells were washed three times in distilled water (dH2O) and then dehydrated with a graded ethanol series: 25% ethanol (1 × 10 min); 50% ethanol (1 × 10 min); 75% ethanol (1 × 10 min); 95% ethanol (1 × 10 min); 100% anhydrous ethanol (3 × 30 min). Bacterial cells deposited onto glass substrate were first sputter coated with a thin layer of Au-Pd (Sputter Coater Denton Vacuum Desk V) to allow subsequent morphological characterization using a FEI Nova NanoSEM 450 at an accelerating voltage of 5 kV with Everhart Thornley Detector (ETD) and Through Lens Detector (TLD) at high magnification.

### Lipopolysaccharide (LPS) isolation, purification and characterization

Dried cells from *P. aeruginosa* PAO1 (1 g) and *P. aeruginosa* ATCC 27853 (0.9 g) were extracted by PCP method, *i.e*. by using phenol/chloroform/petroleum ether (2:5:8 v:v:v)^47^. The yields of extracted LPS was found to be 9% and 5% for *P. aeruginosa* PAO 1 and *P. aeruginosa* ATCC 27853, respectively. Both extracts were analysed by 14% DOC-PAGE, that was performed by using Laemmli procedure^48,49^ and sodium deoxycholate (DOC) as detergent. LPS bands were visualized by silver staining as described previously^50^. The glycosyl analysis was performed as previously reported^51^. Briefly, LPS samples (0.5 mg) were mixed with 1 mL of HCl/CH_3_OH, subjected to methanolysis for 16 hrs at 80 °C, and then acetylated. Simultaneously, another sample of both native LPSs (0.5 mg) was firstly treated with HF (48%; 100 μL) and then subjected to methanolysis and acetylation. Finally, all the acetylated methyl glycosides (MGA) were analysed on an Agilent 7820 A GC System-5977B MSD spectrometer equipped with the automatic injector 7693A and a Zebron ZB-5 capillary column (Phenomenex, Toornace, CA, USA; fow rate 1 mL/min; He as carrier gas). MGA were analysed using the following temperature program: 140 °C for 3 min, 140 °C → 240 °C at 3 °C/min.

### Isothermal titration calorimetry

Interaction between ApoB derived peptides and LPS molecules extracted from *P. aeruginosa* PAO1 or *P. aeruginosa* ATCC 27853 bacterial strains was tested by isothermal titration calorimetry (ITC) experiments, which were carried out on a Low Volume NanoITC (TA instruments, Waters LLC, New Castle, USA) at 37 °C. To this purpose, LPS molecules were diluted to 0.5 mg/mL in 50% phosphate buffer (PBS), and vortexed for 5 min. Afterwards 190 µL of LPS suspension were added to the cell chamber. The syringe was then filled with 50 µL of 266 µM peptide solutions in 50% PBS. Titrations were incremental with 2 µL injections at 300 seconds intervals. Control spectra, obtained by injection of the same amount of each peptide in buffer solution, were subtracted to correct for heat production upon peptide dilution. Collected data were analyzed by using Nano Analyze software (TA instruments, Waters LLC, New Castle, USA).

### Statistical analysis

Statistical analysis was performed using a Student’s t-Test. Significant differences were indicated as *(P < 0.05), **(P < 0.01) or ***(P < 0.001).

## Supplementary information

Effects of human antimicrobial cryptides identified in apolipoprotein B depend on specific features of bacterial strains

## Data Availability

All the data supporting the conclusions have been included within the article.

## References

[1] Agerberth B (1995). FALL-39, a putative human peptide antibiotic, is cysteine-free and expressed in bone marrow and testis. Proc. Natl. Acad. Sci. USA.

[2] Hancock REW, Sahl HG (2006). Antimicrobial and host-defense peptides as new anti-infective therapeutic strategies. Nat. Biotechnol..

[3] Hancock REW (2005). Mechanisms of action of newer antibiotics for Gram-positive pathogens. Lancet Infect. Dis..

[4] Ostorhazi E, Nemes-Nikodem É, Knappe D, Hoffmann R (2014). *In vivo* activity of optimized apidaecin and oncocin peptides against a multiresistant, KPC-producing Klebsiella pneumoniae strain. Protein Pept. Lett..

[5] Steinstraesser L, Kraneburg U, Jacobsen F, Al-Benna S (2011). Host defense peptides and their antimicrobial-immunomodulatory duality. Immunobiology.

[6] Holzl MA, Hofer J, Steinberger P, Pfistershammer K, Zlabinger GJ (2008). Host antimicrobial proteins as endogenous immunomodulators. Immunol. Lett..

[7] Hancock REW, Chapple DS (1999). Peptide antibiotics. Antimicrob. Agents Chemother..

[8] Papo N, Shai Y (2005). Host defense peptides as new weapons in cancer treatment. Cell. Mol. Life Sci..

[9] Barns KJ, Weisshaar JC (2013). Real-time attack of LL-37 on single Bacillus subtilis cells. Biochim Biophys Acta.

[10] Zhang L, Rozek A, Hancock REW (2001). Interaction of cationic antimicrobial peptides with model membranes. J. Biol. Chem..

[11] Yeamen MR, Yount NY (2003). Mechanisms of antimicrobial peptide action and resistance. Pharmacol. Rev..

[12] Joyce GH, Hammond RK, White DC (1970). Changes in membrane lipid composition in exponentially growing Staphylococcus aureus during the shift from 37 to 25 °C. J. Bacteriol..

[13] Bozdogan B, Esel D, Whitener C, Browne FA, Appelbaum PC (2003). Antibacterial susceptibility of a vancomycin-resistant Staphylococcus aureus strain isolated at the Hershey Medical Center. J. Antimicrob. Chemother..

[14] Lee MT, Hung WC, Chen FY, Huang HW (2008). Mechanism and kinetics of pore formation in membranes by water soluble amphipathic peptides. Proc. Natl. Acad. Sci. USA.

[15] Biaggi MH, Riske KA, Lamy-Freund MT (1997). Melanotropic peptides-lipid bilayer interaction. Comparison of the hormone alpha-MSH to a biologically more potent analog. Biophys. Chem..

[16] Biaggi MH, Pinheiro TJ, Watts A, Lamy-Freund MT (1996). Spin label and 2H-NMR studies on the interaction of melanotropic peptides with lipid bilayers. Eur. Biophys. J..

[17] Ramamoorthy A, Thennarasu S, Lee DK, Tan A, Maloy L (2006). Solid-state NMR investigation of the membrane-disrupting mechanism of antimicrobial peptides MSI-78 and MSI-594 derived from magainin 2 and melittin. Biophys. J..

[18] Henzler Wildman KA, Lee DK, Ramamoorthy A (2003). Mechanism of lipid bilayer disruption by the human antimicrobial peptide, LL-37. Biochemistry.

[19] Hong M, Su Y (2011). Structure and dynamics of cationic membrane peptides and proteins: Insights from solid-state NMR. Protein Sci..

[20] Gaglione R (2017). Novel human bioactive peptides identified in Apolipoprotein B: Evaluation of their therapeutic potential. Biochem. Pharmacol..

[21] Pane K (2017). Antimicrobial potency of cationic antimicrobial peptides can be predicted from their amino acid composition: Application to the detection of “cryptic” antimicrobial peptides. J. Theor. Biol..

[22] Gaglione R (2017). Insights into the anticancer properties of the first antimicrobial peptide from Archaea. Biochim Biophys Acta Gen Subj.

[23] Bosso A (2017). A new cryptic host defense peptide identified in human 11-hydroxysteroid dehydrogenase-1 β-like: from in silico identification to experimental evidence. Biochim Biophys Acta Gen Subj.

[24] Zanfardino A (2018). Human apolipoprotein E as a reservoir of cryptic bioactive peptides: The case of ApoE 133-167. J. Pept. Sci..

[25] Pizzo Elio, Pane Katia, Bosso Andrea, Landi Nicola, Ragucci Sara, Russo Rosita, Gaglione Rosa, Torres Marcelo D.T., de la Fuente-Nunez Cesar, Arciello Angela, Di Donato Alberto, Notomista Eugenio, Di Maro Antimo (2018). Novel bioactive peptides from PD-L1/2, a type 1 ribosome inactivating protein from Phytolacca dioica L. Evaluation of their antimicrobial properties and anti-biofilm activities. Biochimica et Biophysica Acta (BBA) - Biomembranes.

[26] Kasetty G (2011). The C-terminal sequence of several human serine proteases encodes host defense functions. J. Innate Immun..

[27] Lee DY (2009). Histone H4 is a major component of the antimicrobial action of human sebocytes. J. Invest. Dermatol..

[28] Beck WH (2013). Apolipoprotein A-I binding to anionic vesicles and lipopolysaccharides: role for lysine residues in antimicrobial properties. Biochim Biophys Acta.

[29] van Dijk A (2009). Chicken heterophils are recruited to the site of Salmonella infection and release antibacterial mature Cathelicidin-2 upon stimulation with LPS. Mol. Immunol..

[30] MacGowan AP (1996). A new time-kill method of assessing the relative efficacy of antimicrobial agents alone and in combination developed using a representative beta-lactam, aminoglycoside and fluoroquinolone. J. Antimicrob. Chemother..

[31] Dhariwal AK, Tullu MS (2013). Colistin: re-emergence of the ‘forgotten’ antimicrobial agent. J. Postgrad. Med..

[32] González C, Rubio M, Romero-Vivas J, González M, Picazo JJ (1999). Bacteremic pneumonia due to Staphylococcus aureus: A comparison of disease caused by methicillin-resistant and methicillin-susceptible organisms. Clin. Infect. Dis..

[33] Arsenault TL (1991). Structural studies on the polysaccharide portion of A-band lipopolysaccharide from a mutant (AK1401) of Pseudomonas aeruginosa strain PAO1. Can J Chem.

[34] Sadovskaya I (2000). Structural characterization of the outer core and the O-chain linkage region of lipopolysaccharide from Pseudomonas aeruginosa serotype O5. Eur. J. Biochem..

[35] Fernandes P, Martens E (2017). Antibiotics in late clinical development. Biochem. Pharmacol..

[36] Halder S (2015). Alteration of Zeta potential and membrane permeability in bacteria: a study with cationic agents. Springerplus.

[37] Yadavalli SS (2016). Antimicrobial peptides trigger a division block in Escherichia coli through stimulation of a signalling system. Nat Commun.

[38] Lutkenhaus J (1990). Regulation of cell division in E. coli. Trends Genet..

[39] Salomón RA, Farías RN (1992). Microcin 25, a novel antimicrobial peptide produced by Escherichia coli. J. Bacteriol..

[40] Ishikawa M, Kubo T, Natori S (1992). Purification and characterization of a diptericin homologue from Sarcophaga peregrina (flesh fly). Biochem. J..

[41] Chileveru HR (2015). Visualizing attack of Escherichia coli by the antimicrobial peptide human defensin 5. Biochemistry.

[42] Gaspar D, Veiga AS, Sinthuvanich C, Schneider JP, Castanho MA (2012). Anticancer peptide SVS-1: efficacy precedes membrane neutralization. Biochemistry.

[43] Vincent JL, Opal SM, Marshall JC, Tracey KJ (2013). Sepsis definitions: time for change. Lancet.

[44] Bommarius B (2010). Cost-effective expression and purification of antimicrobial and host defense peptides in Escherichia coli. Peptides.

[45] Gaglione R (2019). Cost-effective production of recombinant peptides in Escherichia coli. N. Biotechnol..

[46] Helander IM, Mattila-Sandholm T (2000). Fluorometric assessment of gram-negative bacterial permeabilization. J. Appl. Microbiol..

[47] Galanos C, Lüderitz O, Westphal O (1969). A new method for the extraction of R lipopolysaccharides. Eur. J. Biochem..

[48] Laemmli UK (1970). Most commonly used discontinuous buffer system for SDS electrophoresis. Nature.

[49] Carillo S (2014). Structural investigation of the antagonist LPS from the cyanobacterium Oscillatoria planktothrix FP1. Carbohydr. Res..

[50] Tsai CM, Frasch CE (1982). A sensitive silver stain for detecting lipopolysaccharides in polyacrylamide gels. Anal. Biochem..

[51] Pieretti G (2010). The complete structure of the core of the LPS from Plesiomonas shigelloides 302-73 and the identification of its O-antigen biological repeating unit. Carbohydr. Res..

