# Supplementary material for: Effects of human antimicrobial cryptides identified in apolipoprotein B depend on specific features of bacterial strains
Source: Sci Rep. 2019 Apr 30;9:6728. doi: 10.1038/s41598-019-43063-3 (PMC6491590; doi:10.1038/s41598-019-43063-3)
Supplement: Supplementary file 1 — Effects of human antimicrobial cryptides identified in apolipoprotein B depend on specific features of bacterial strains [file 41598_2019_43063_MOESM1_ESM.pdf]

Supplementary Info

Effects of human antimicrobial cryptides identified in apolipoprotein B depend on specific features of bacterial strains

Rosa Gaglione[1], Angela Cesaro[1], Eliana Dell'Olmo[1], Bartolomeo Della Ventura[2], Angela Casillo[1], Rocco Di Girolamo[1], Raffaele Velotta[2], Eugenio Notomista[3], Edwin J.A. Veldhuizen[4], Maria Michela Corsaro[1], Claudio De Rosa[1], Angela Arciello[1,5,*]

[1]Department of Chemical Sciences, University of Naples Federico II, 80126 Naples, Italy; [2]Department of Physics, University of Naples Federico II, 80126 Naples, Italy; [3]Department of Biology, University of Naples Federico II, 80126 Naples, Italy; [4]Department of Infectious Diseases and Immunology, Division Molecular Host Defence, Faculty of Veterinary Medicine, Utrecht University, Utrecht, The Netherlands; [5]Istituto Nazionale di Biostrutture e Biosistemi (INBB), Italy.

***Correspondence**: Dr. Angela Arciello, Department of Chemical Sciences, University of Naples Federico II, Via Cintia 4, 80126 Naples, Italy (email: anarciel@unina.it).

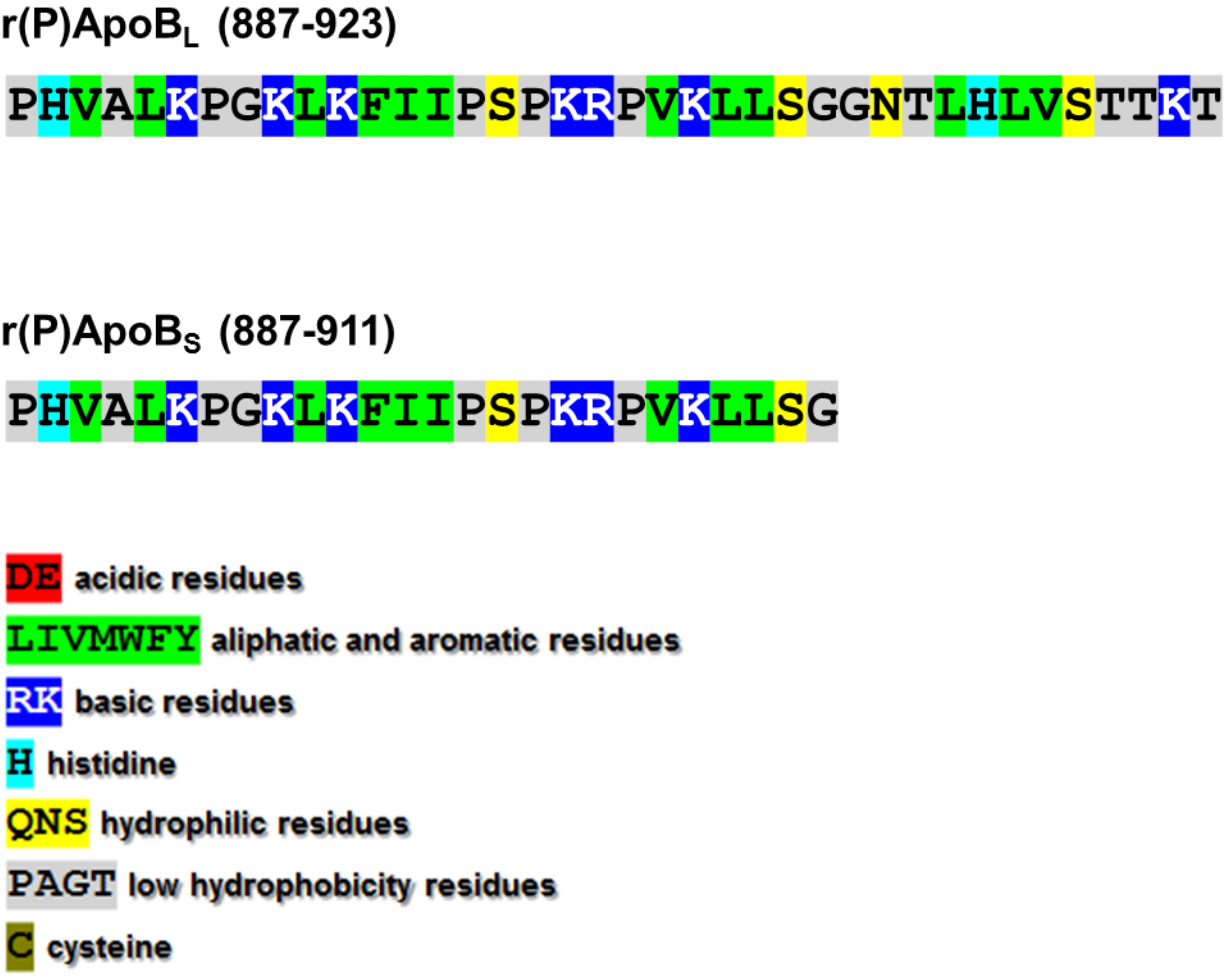

Figure S1. ApoB derived peptides primary structure. Color code refers to amino acids properties.

Table S1. **Minimum Inhibitory Concentration (MIC, µM)** values determined for r(P)ApoB$_L$ and r(P)ApoB$_S$ peptides tested on a panel of Gram-negative and Gram-positive bacterial strains.

	**MIC$_{100}$ (µM)**	
	**r(P)ApoB$_L$**	**r(P)ApoB$_S$**
**Gram-negative strains**		
P. aeruginosa PAO1	20	20
P. aeruginosa ATCC 27853	> 40	> 40
**Gram-positive strains**		
B. globigii TNO BM013	5	2.5
S. aureus MRSA WKZ-2	> 40	> 40

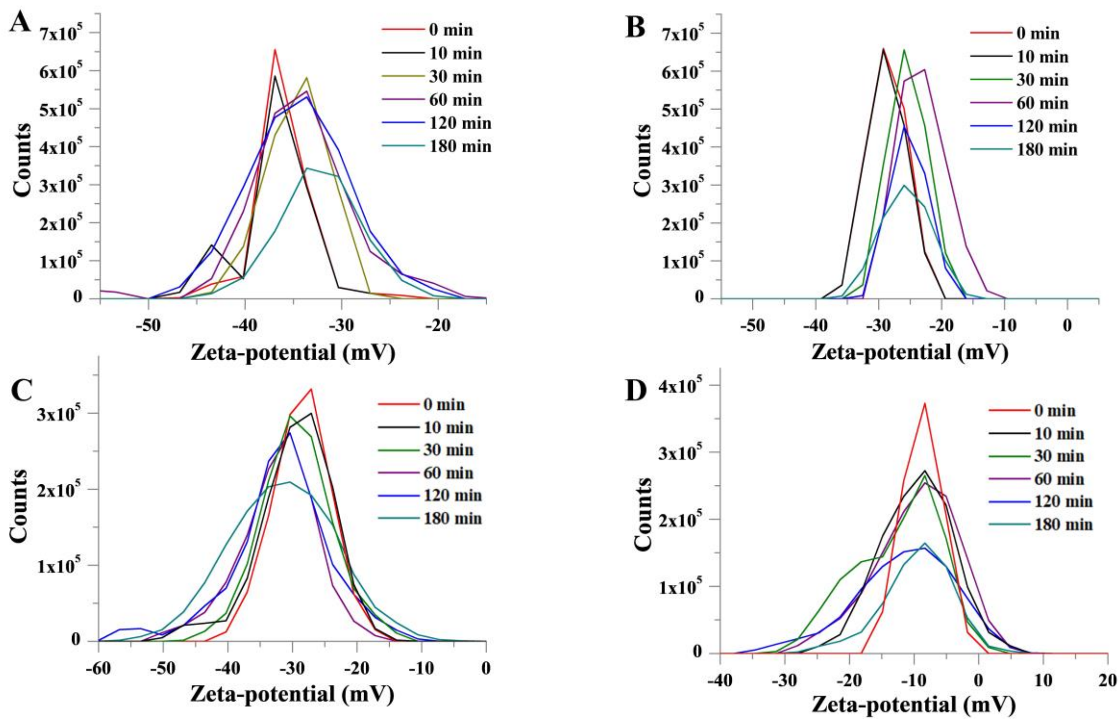

Figure S2. Zeta-potential values of bacterial cells. Zeta-potential values recorded for *B. globigii* TNO BM013 (A), *S. aureus* MRSA WKZ-2 (B), *P. aeruginosa* PAO1 (C), and *P. aeruginosa* ATCC 27853 (D) in culture medium (NB 0.5X) are reported over time (0-180 min). Data represent the average of at least three independent experiments.

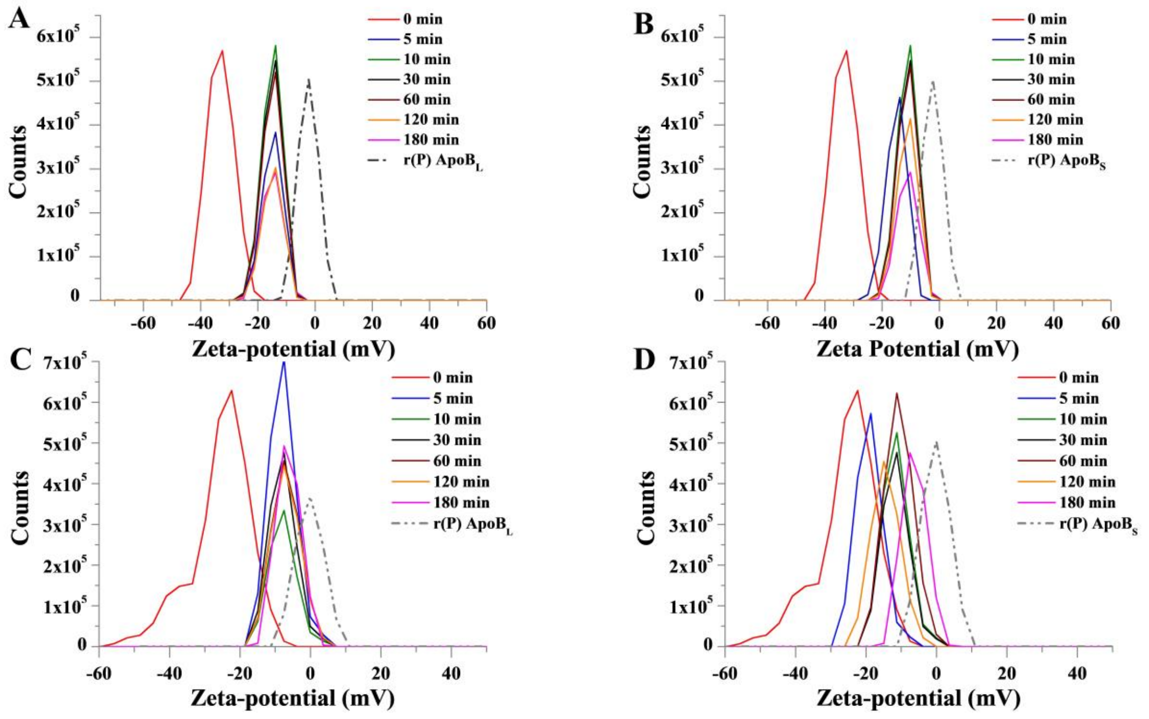

Figure S3. ApoB derived peptides effects on the Zeta-potential of treated bacterial cells. Zeta-potential values of *B. globigii* TNO BM013 (A, B) and *P. aeruginosa* PAO1 (C, D) bacterial strains were determined upon treatment with r(P)ApoB$_L$ (A, C) or r(P)ApoB$_S$ (B, D) peptides at a concentration corresponding to MIC$_{100}$ values. Zeta-potential values were recorded at regular time intervals for 180 min. Dashed curves represent the Zeta-potential values of ApoB derived peptides alone in bacterial culture medium (NB 0,5X). Data represent the average of at least three independent experiments.

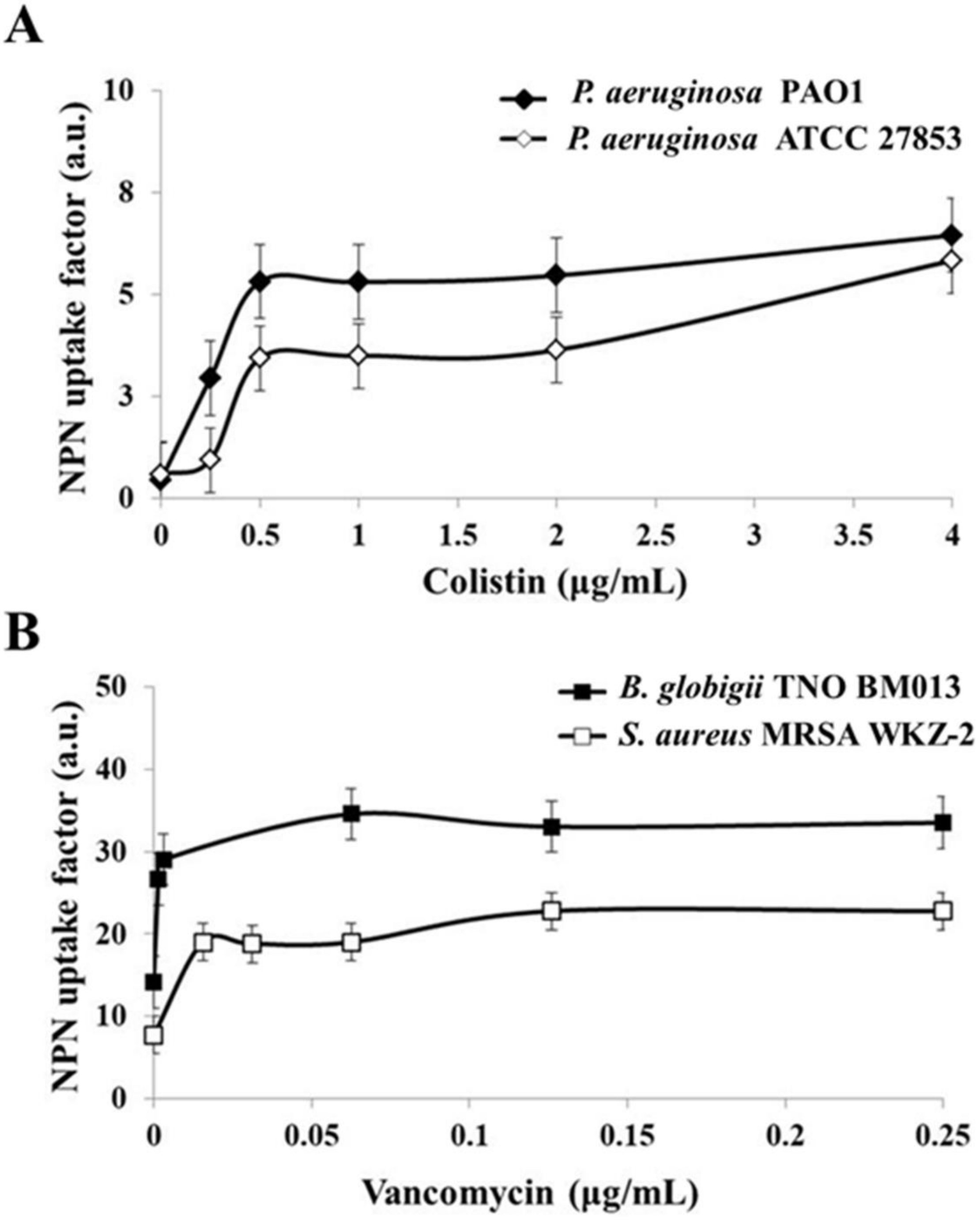

Figure S4. Effects of increasing concentrations of colistin and vancomycin antibiotics on bacterial membrane permeability. NPN uptake factors were evaluated upon treatment of *B. globigii* TNO BM013, *P. aeruginosa* PAO1, *S. aureus* MRSA WKZ-2 and *P. aeruginosa* ATCC 27853 bacterial strains with increasing concentrations of colistin (0.25 - 4 µg/mL) (A) and vancomycin (0.05 – 0.25 µg/mL) (B). Values derive from a minimum of three independent experiments, each one carried out with triplicate determinations.

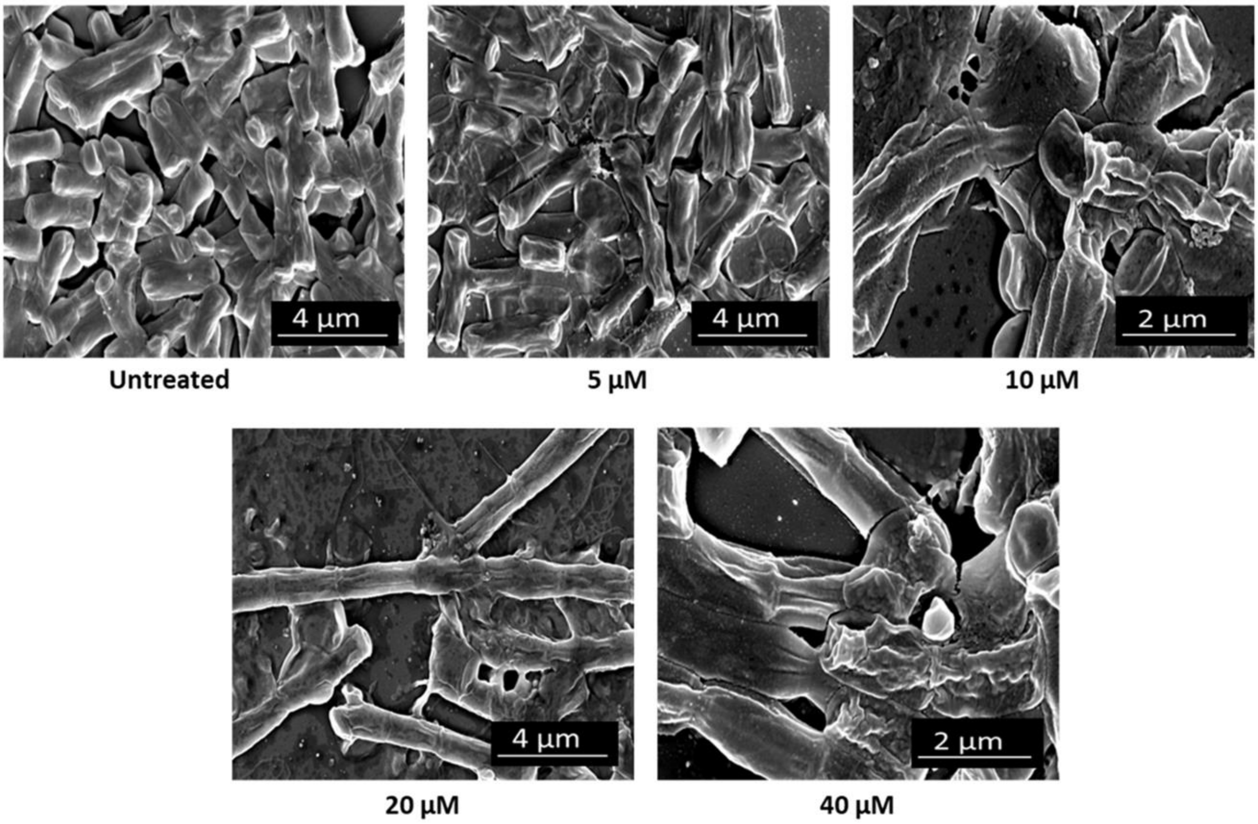

Figure S5. Morphological analyses of *B. globigii* TNO BM013 cells by SEM. Representative images are shown upon treatment of bacterial cells with increasing concentrations (5 - 40 µM) of r(P)ApoB$_L$. A total of 60 cells were analysed for each sample in two independent experiments. Bars 2 or 4 µm.

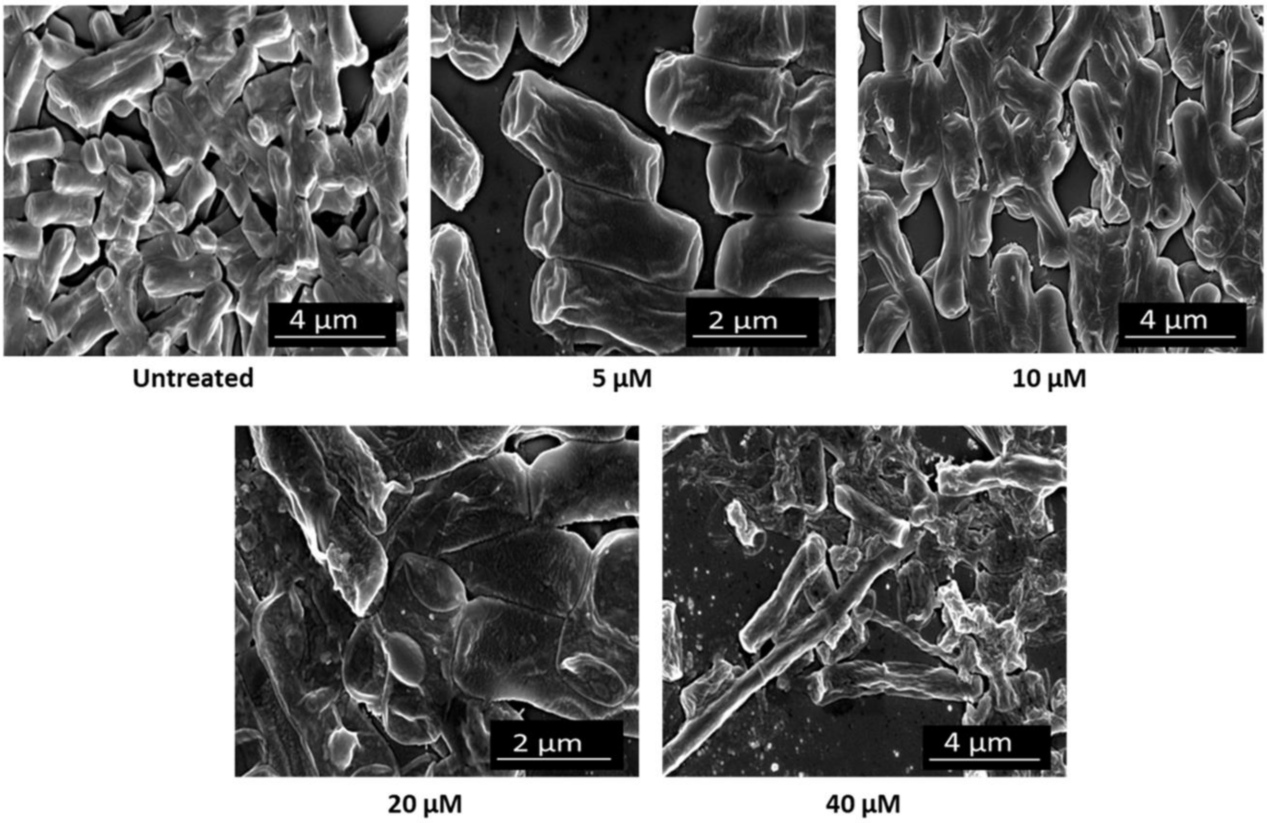

Figure S6. Morphological analyses of *B. globigii* TNO BM013 cells by SEM. Representative images are shown upon treatment of bacterial cells with increasing concentrations (5 - 40 µM) of r(P)ApoB$_S$. A total of 60 cells were analysed for each sample in two independent experiments. Bars 2 or 4 µm.

Table S2. Biophysical characterization of the binding of ApoB derived peptides to LPS molecules extracted from *P. aeruginosa* PAO1 and *P. aeruginosa* ATCC 27853 bacterial strains.

**PARAMETERS**	LPS from *P. aeruginosa* PAO1		LPS from *P. aeruginosa* ATCC 27853	
	**r(P)ApoB$_L$**	**r(P)ApoB$_S$**	**r(P)ApoB$_L$**	**r(P)ApoB$_S$**
**Enthalpy (KJ/mol)**	51.08	42.82	52.07	47
**Entropy (J/mol)**	269	244	272	260
**Dissociation constant, K_d (M)**	3.58×10^{-6}	2.94×10^{-6}	3.56×10^{-6}	2.09×10^{-6}
**Stoichiometry (n)**	0.65	0.50	0.43	0.35
